# Supplementary material for: The rise of South–South trade and its effect on global CO2 emissions
Source: Nat Commun. 2018 May 14;9:1871. doi: 10.1038/s41467-018-04337-y (PMC5951843; doi:10.1038/s41467-018-04337-y)
Supplement: Supplementary file 1 — Supplementary Information [file 41467_2018_4337_MOESM1_ESM.pdf]

Supporting Information

The rise of South-South trade and its effect on global CO_2 emissions

Jing Meng et al.

Supplementary Table 1. Emissions embodied in international trade

	2004 (Gt CO_2)	2007 (Gt CO_2)	Annual growth rate 2004-2007	2011 (Gt CO_2)	Annual growth rate 2007-2011
From developing regions to developed regions	1.8	2.0	3.6%	2.2	2.4%
From developing regions to developing regions	0.47	0.72	15.3%	1.12	12%
China's exported emissions	1.03	1.41	11%	1.74	5%
India's exported emissions	0.16	0.2	7.7%	0.32	12%
**Total emissions embodied in developing regions' exports emissions**	2.2	2.8	8.4%	3.3	4%

Supplementary Table 2. Coal share and emission intensity in China and India

	2004	2005	2006	2007	2008	2009	2010	2011	2012	2013	2014	2015
India [a]	49.9%	53.7%	53.0%	53.3%	54.5%	54.9%	52.3%	53.2%	55.0%	56.8%	58.3%	58.1%
China [b]	70.2%	72.4%	72.4%	72.5%	71.5%	71.6%	69.2%	70.2%	69%	67%	66%	64%
China's emission intensity (kg/$US)	2.58	2.64	2.59	2.49	2.33	2.27	2.25	2.26	2.14	2.06	1.94	1.89
India's emission intensity (kg/$US) [b]	1.62	1.56	1.54	1.56	1.58	1.62	1.57	1.58	1.59	1.55	1.57	1.54

Data source: [a] India's coal share (BP Statistical Review of World Energy, 2015, 2016); India's CO_2 emissions (international Energy Agency); India's GDP in constant 2000 price (National Account Main Aggregates Database); China's GDP, coal share, CO_2 emissions (CEADS database, http://www.ceads.net/)

Supplementary Table 3. Abbreviation and concordance of world regions.

World region	Abbreviation	GTAP regions	Notes
China	China	4	Developing regions
Economies in Transition (Eastern Europe and former Soviet Union)	EIT	52,54,59,62,63,67,69, 70,78,79,80,81,82,83, 84,85,86,87,88,89,90, 91	
Latin America and Caribbean	LAM	28,29,30,31,32,33,34, 35,36,37,38,39,40,41, 42,43,44,45,46,47,48	Developing regions
Middle East and North Africa	MNA	92,93,94,95,96,97,98, 99,100,101,102,103,1 04,105	Developing regions
North America (USA, Canada)	NAM	26,27	
Developed regions in Asia and Pacific	POECD	1,2,5,6,7,9,16	
Developing regions in Asia and Pacific	OAS	3,8,10,11,12,13,14,15, 17,18,19,20,22,23,24, 25	Developing regions
India	India	21	Developing regions
sub-Saharan Africa	SSA	106,107,109,108,110, 111,112,113,114,115, 116,117,118,119,120, 121,122,123,124,125, 126,127,128,12	Developing regions
Western Europe	WEU	49,50,51,53,55,56,57, 58,60,61,64,65,66,68, 71,72,73,74,75,76,77	

Supplementary Table 4. Definition of sectors.

Number	Sector	Category
1	Paddy rice	Agriculture
2	Wheat	
3	Cereal grains nec	
4	Vegetables, fruit, nuts;	
5	Oil seeds	
6	Sugarcane, sugar beet	
7	Plant-based fibers	
8	Crops nec	
9	Cattle, sheep, goats, horses	
10	Animal products nec	
11	Raw milk	
12	Wool, silk-worm cocoons	
13	Forestry	
14	Fishing	
15	Coal	Mining
16	Oil	
17	Gas	
18	Minerals nec	
19	Meat: cattle, sheep, goats, horses	Light industry
20	Meat products nec	
21	Vegetable oils and fats	
22	Dairy products	
23	Processed rice	
24	Sugar	
25	Food products nec	
26	Beverages and tobacco products	
27	Textiles	
28	Wearing apparel	
29	Leather products	
30	Wood products	
31	Paper products, publishing	
32	Petroleum, coal products	Heavy industry
33	Chemical, rubber, plastic products	
34	Mineral products nec	
35	Ferrous metals	
36	Metals nec	
37	Metal products	
38	Motor vehicles and parts	
39	Transport equipment nec	
40	Electronic equipment	Hi-tech
41	Machinery and equipment nec	

42	Manufactures nec	
43	Electricity	Heavy industry
44	Gas manufacture, distribution	
45	Water collection, purification, and distribution	
46	Construction	Construction
47	Trade	Service
48	Transport nec	
49	Sea transport	
50	Air transport	
51	Communication	
52	Financial services nec	
53	Insurance	
54	Business services nec	
55	Recreation and other services	
56	Public Administration, Defense, Health, Education	
57	Dwellings	

Supplementary Table 5. Sector classification of pricing data for global MRIO tables.

	**Sectors**	**Sector classification**	**GTAP sectors**
1	Agriculture, hunting, forestry, fishing	ISIC A-B	1-14
2	Mining, Manufacturing, Utilities	ISIC C-E	15-42
3	Manufacturing	ISIC D	43-45
4	Construction	ISIC F	46
5	Wholesale, retail trade, restaurants and hotels	ISIC G-H	47
6	Transport, storage and communication	ISIC I	48-51
7	Other Activities	ISIC J-P	52-57

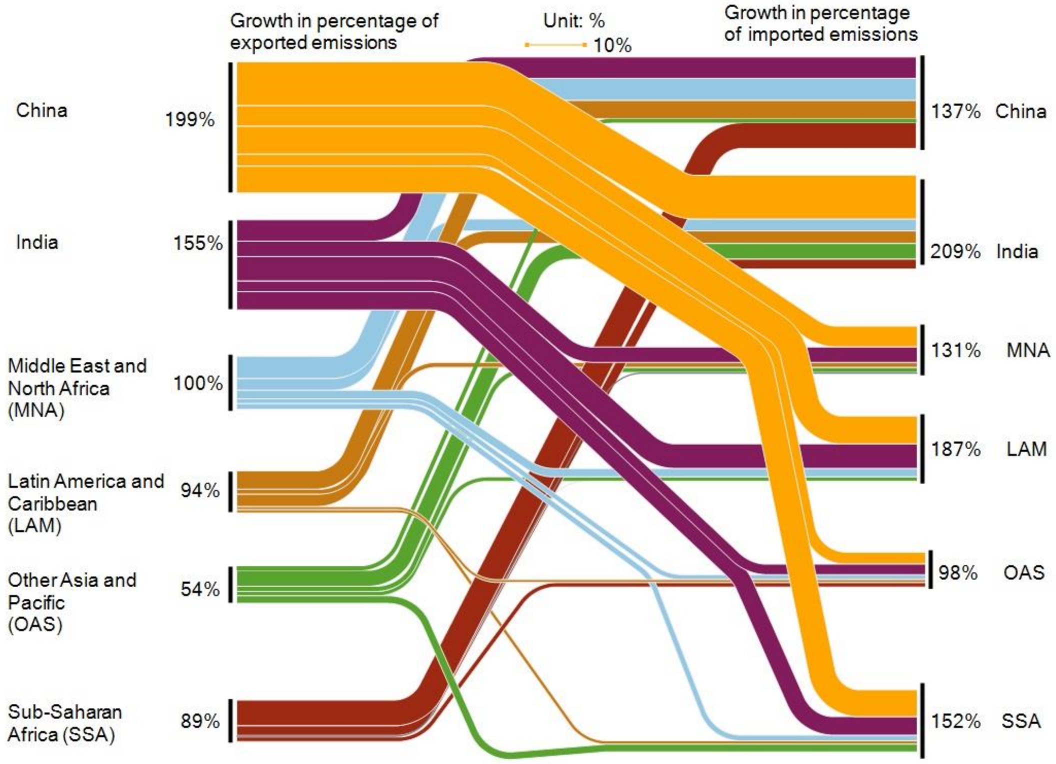

Supplementary Fig. 1| Percentage change in bilateral emission transfers (from fossil fuel combustion) among world regions from 2004 to 2011 compared with 2004 (unit: %). Flow width representing the percentage change in bilateral emission transfers (in %) between 2004 and 2011 compared with 2004 (from left-hand region to right-hand region). Percentages on the left-hand side indicate the increased total exported emissions per exporter to the regions in the right side from 2004 to 2011 compared with 2004, whereas percentages on the right-hand side indicate the increases in total imported emissions per importer from 2004 to 2011 compared with 2004. The negative flow value is depicted in gray.

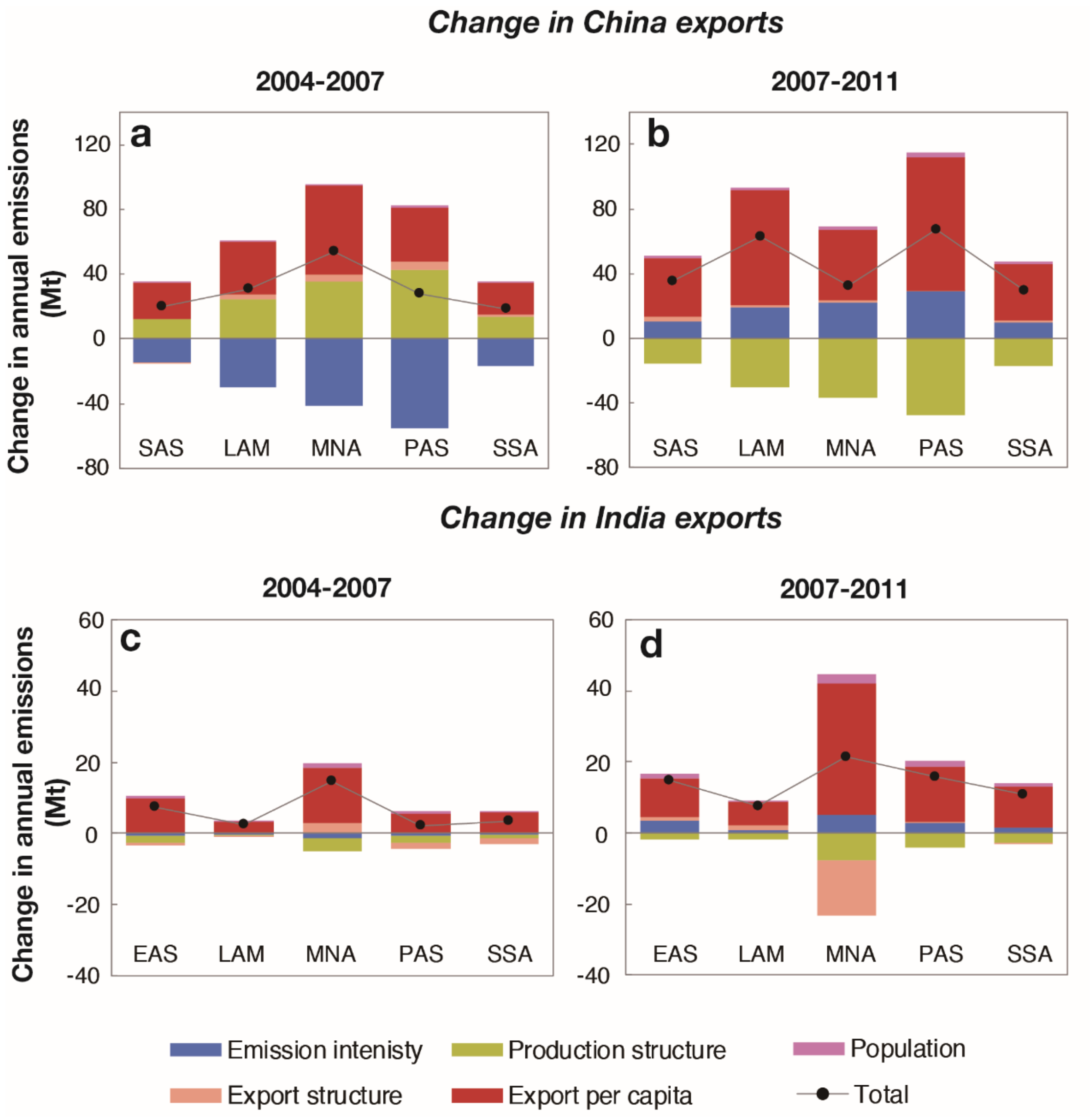

Supplementary Fig. 2 | Contributions of different factors to changes in China's and India's exports during 2004-2007 and 2007-2011.

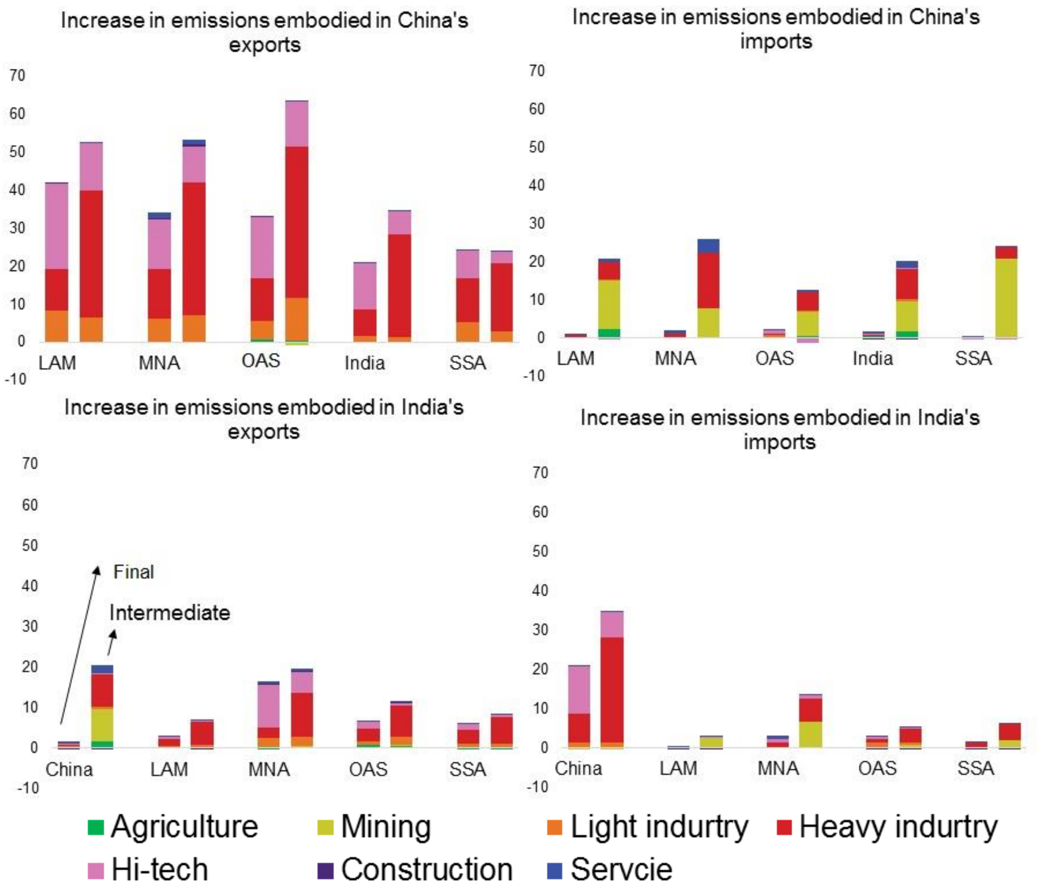

Supplementary Fig. 3|Change in emissions embodied in trade between developing regions from 2004 to 2011 in sectoral details. Bars show the balance of CO_2 emissions embodied in trade between developing regions and China (top panel) and India (bottom panel). Colors indicate the sectors of traded products, with final and intermediate products differentiated by separate bars. The details of sector aggregation are shown in Table S4.

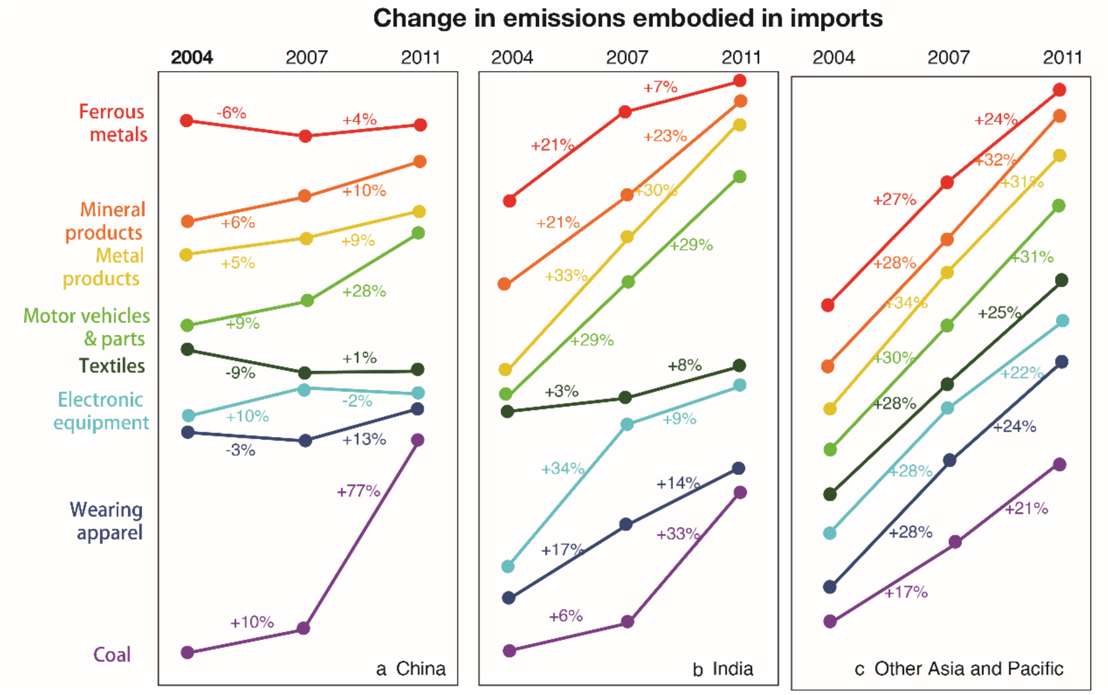

Supplementary Fig. 4 | Annual changes in emissions embodied in selected types of imported products from the rest of World. Lines show relative changes in the emissions embodied in different types of products exported by China (**a**), India (**b**) and Other Asia and Pacific region (**c**).
