# Supplementary material for: The rise of South–South trade and its effect on global CO2 emissions
Source: Nat Commun. 2018 May 14;9:1871. doi: 10.1038/s41467-018-04337-y (PMC5951843; doi:10.1038/s41467-018-04337-y)
Supplement: Supplementary file 3 — Description of Additional Supplementary Information [file 41467_2018_4337_MOESM3_ESM.docx]

**Description of Additional Supplementary Files**

File Name: Supplementary Data 1

Description: annual changes in emissions embodied in exports in selected regions.

File Name: Supplementary Data 2

Description: definition of regions.

File Name: Supplementary Data 3

Description: South-South transfers of embodied emissions in 2004, 2007 and 2011.
